# Adverse events associated with bidirectional endoscopy with midazolam and pethidine

**DOI:** 10.3164/jcbn.19-73

**Published:** 2019-10-30

**Authors:** Toshihiro Nishizawa, Kosuke Sakitani, Hidekazu Suzuki, Mami Takeuchi, Yoshiyuki Takahashi, Kazue Takeuchi, Tadahiro Yamakawa, Shuntaro Yoshida, Keisuke Hata, Hirotoshi Ebinuma, Kazuhiko Koike, Osamu Toyoshima

**Affiliations:** 1 Gastroenterology, Toyoshima Endoscopy Clinic, 6-17-5 Seijo, Setagaya-ku, Tokyo 157-0066, Japan; 2 Department of Gastroenterology and Hepatology, International University of Health and Welfare, Mita Hospital, 1-4-3 Mita, Minato-ku, Tokyo 108-8329, Japan; 3 Gastroenterology, Sakitani Endoscopy Clinic, 7-7-1 Yazu, Narashino city, Chiba 275-0026, Japan; 4 Department of Gastroenterology and Hepatology, Tokai University School of Medicine, 143 Shimokasuya, Isehara city, Kanagawa 259-1193, Japan; 5 Department of Gastroenterology, Graduate School of Medicine, The University of Tokyo, 7-3-1 Hongo, Bunkyo-ku, Tokyo 113-0033, Japan; 6 Department of Surgical Oncology, Graduate School of Medicine, The University of Tokyo, 7-3-1 Hongo, Bunkyo-ku, Tokyo 113-0033, Japan

**Keywords:** bidirectional endoscopy, hypoxia, nausea, pethidine

## Abstract

Same-day bidirectional endoscopy has been reported to reduce recovery time, and procedure-related cost. The safety of bidirectional endoscopy vs colonoscopy only, while using midazolam and pethidine, has never been evaluated. We reviewed 1,202 consecutive patients who underwent bidirectional endoscopy or colonoscopy only with administration of midazolam and pethidine in Toyoshima Ensdoscopy Clinic. We compared the clinical characteristics and adverse events associated with method of endoscopy (colonoscopy only vs bidirectional endoscopy). Furthermore, multivariate logistic regression analyses were conducted to study the role of age, sex, use of sedative, polypectomy, and bidirectional endoscopy in adverse events. In the bidirectional endoscopy group, the doses of pethidine and midazolam, and the incidence rates of hypoxia and posto-endoscopic nausea were significantly higher. On multivariate analysis, age (odds ratio = 1.061, *p*<0.001), use of pethidine (odds ratio = 4.311, *p* = 0.003), and bidirectional endoscopy (odds ratio = 3.658, *p*<0.001) were independently associated with hypoxia. On multivariate analysis, female sex (odds ratio = 10.25, *p* = 0.027) and bidirectional endoscopy (odds ratio = 6.051, *p* = 0.022) were independently associated with post-endoscopic nausea. In conclusion, bidirectional endoscopy could increase hypoxia in elderly patients using pethidine and post-endoscopic nausea in female patients.

## Introduction

The combination of esophagogastroduodenoscopy (EGD) and colonoscopy is commonly performed in the endoscopic unit to evaluate patients including those with positive fecal occult blood test and iron-deficiency anemia for gastrointestinal bleeding.^(1,2)^ The combination of EGD and colonoscopy, which is termed bidirectional endoscopy, is also indicated in asymptomatic patients during physical check-up or cancer screening.^(3)^

Lucendo *et al.*^(4)^ reported that the same-day bidirectional endoscopy with non-anesthesiologist administration of propofol resulted in reductions in propofol doses, recovery time, and procedure-related cost as compared with EGD and colonoscopy performed separately, without an increase in adverse events. However, propofol is not always available, because the manufacturers of propofol restrict its use solely to personnel trained in general anesthesia.^(5,6)^ On the other hand, benzodiazepines such as midazolam are the most commonly used sedative, and these are generally given with an opioid analgesic such as pethidine or fentanyl for synergistic effect. The safety of bidirectional endoscopy vs colonoscopy only, while using midazolam and pethidine, has never been evaluated. This study evaluated the safety of same-day bidirectional endoscopy with administration of midazolam and pethidine.

## Methods

### Ethics

This retrospective study was approved by the Ethical Review Committee of the Hattori Clinic on September 7, 2018. Written informed consent was obtained from the study participants.^(7,8)^ All clinical investigations were conducted according to the ethical guidelines of the Declaration of Helsinki.

### Subjects

This study included subjects who agreed to participate in the study and underwent same-day bidirectional endoscopy or colonoscopy only at Toyoshima Endoscopy Clinic, an outpatient clinic specialized in endoscopy, between May 1, 2017 and August 31, 2017. Bidirectional endoscopy or colonoscopy only was performed to evaluate patients with gastrointestinal bleeding, including patients with positive results for the fecal occult blood test, iron-deficiency anemia, and abdominal pain and those who had undergone screening for cancer, polyps, atrophic gastritis, and physical check-up. The following demographic and clinical characteristics were collected from medical records: age, sex, doses of midazolam and pethidine, and adverse events. Adverse events included hypoxia during and after procedures and post-endoscopic nausea, abdominal pain, and gastrointestinal bleeding and perforation. Hypoxia was defined as reduction in oxygen saturation <90% for more than 20 s or implementation of oxygen inhalation based on the judgement of the on-site endoscopist. The diagnostic criteria for post-endoscopic nausea included nausea or vomiting within 24 h after endoscopy.

### Endoscopic examination

Patients, who participated in the study underwent colonic preparation using 2 L of polyethylene glycol solution administered orally 5 h before the procedure. Polyethylene glycol solution or magnesium citrate was added when the stool was not clear liquid.^(9)^ Bidirectional endoscopy or colonoscopy only was performed by experienced endoscopists. The sequence for bidirectional endoscopy is EGD followed by colonoscopy. Before starting EGD, the pharynx of patients was topically anesthetized by gargling with 2% lidocaine hydrochloride viscous solution (Xylocaine Viscous 2%, AstraZeneca Inc., Japan).^(10)^ Sedation with midazolam and/or pethidine was induced based on the patient’s willingness.^(11)^ Endoscopists were allowed to use clinical judgement in deciding the dose and type of sedative and analgesic medication; midazolam (0–10 mg) and pethidine (0–87.5 mg). For elderly patients (age ≥70 years), reduced doses of sedative were used.^(12)^ For insufflation, CO_2_ was administered in the absence of chronic respiratory failure. Colonoscope insertion in the cecum was accomplished using standard maneuvers. In the absence of contraindications, when the colonoscope reached the cecum, we administered 10 to 20 mg of scopolamine butylbromide. Lesions, diagnosed as adenomas or sessile serrated polyps, were removed by using hot or cold polypectomy with a snare or forceps or by endoscopic mucosal resection on the examination day. We did not resect the polyps with a diameter of 20 mm or more because they should be resected in the hospitalization facility.^(13)^

Patients were transferred to the recovery room after the procedure. All adverse events including hypoxia and nausea were evaluated by the recovery room nurse. Patients were requested to return for a follow up visit 10 to 14 days after the procedure for an explanation of the endoscopic findings, and they were also counselled for adverse events.^(14)^

### Statistical analysis

We compared the clinical characteristics and adverse events between bidirectional endoscopy and colonoscopy only with the use of Welch’s *t* test or chi-square test. If a zero frequency arose in the chi-square test, 0.5 was added to all of the cells of a fourfold table to estimate odds ratio (Haldane correction).^(15)^

The clinical parameters were analyzed using univariate logistic regression analysis. The predictors found to be associated with hypoxia or post-endoscopic nausea on univariate analysis (*p*<0.1) were subsequently assessed using multiple logistic regression method to identify independent factors. Age, body weight, body mass index, and dose per kilogram of body weight of each drug were included as continuous variables in the univariate and multivariate logistic regression methods. A *p* value of less than 0.05 was considered statistically significant. The data were analyzed using the Stat Mate IV software (ATOMS, Tokyo, Japan).

## Results

During the study period, 1,202 consecutive patients underwent bidirectional endoscopy or colonoscopy only. The characteristics of bidirectional endoscopy group and colonoscopy only group were shown in Table 1. In bidirectional endoscopy group, age was significantly lower (*p* = 0.0078), and women were significantly less (*p* = 0.025). In the bidirectional endoscopy group, the dose of pethidine and midazolam and occurrence rates of hypoxia and nausea were significantly higher (*p* value <0.001, <0.001, <0.001 and 0.0158 respectively) than those in the colonoscopy only group (Table 1).

Table 2 shows the univariate and multivariate analysis for hypoxia during and after the procedure. On univariate analysis, age (odds ratio = 1.047, *p*<0.001), use of pethidine (odds ratio = 4.692, *p*<0.001), with polypectomy (odds ratio = 2.089, *p* = 0.016), and bidirectional endoscopy (odds ratio = 3.963, *p*<0.001) were significantly associated with hypoxia during and after the procedure. On multivariate analysis, age (odds ratio = 1.061, *p*<0.001), bidirectional endoscopy (odds ratio = 3.658, *p*<0.001), and use of pethidine (odds ratio = 4.311, *p* = 0.003) were independently associated with hypoxia during and after the procedure.

Table 3 shows the univariate and multivariate analysis for post-endoscopic nausea. On univariate analysis, female sex (odds ratio = 9.21, *p* = 0.024) and bidirectional endoscopy (odds ratio =colonoscopy5.406, *p* = 0.035) were significantly associated with post-endoscopic nausea. On multivariate analysis, female sex (odds ratio = 10.25, *p* = 0.027) and bidirectional endoscopy (odds ratio = 6.051, *p* = 0.022) were independently associated with post-endoscopic nausea.

## Discussion

Same-day bidirectional endoscopy with administration of midazolam and pethidine has significantly increased hypoxia and post-endoscopic nausea. Furthermore, old age and use of pethidine are independent risk factors for hypoxia. Female sex is an independent risk factor for post-endoscopic nausea. To the best of our knowledge, this is the first report of increased adverse events in same-day bidirectional endoscopy.

Peri-endoscopic sedatives and analgesics are often used to provide patient comfort and improve examination quality during endoscopic procedures. Benzodiazepines such as midazolam are the most commonly used sedative, and these are generally given to the patient along with an opioid analgesic for synergistic effect. However, respiratory depression is the major adverse effect of sedative use. Bidirectional endoscopy requires more sedation due to longer procedural time resulting in higher incidence of hypoxia. In the elderly patients, EGD and colonoscopy performed separately might be a better choice.

Female sex and bidirectional endoscopy are independently associated with post-endoscopic nausea. In this study, all of the 11 patients who had post-endoscopic nausea used pethidine. Nausea and vomiting might be the result of stimulation of the medullary chemoreceptor trigger zone by pethidine.^(14)^ The observed sex differences can be explained by the presence of a different socialization process for men and women that may influence the willingness to communicate distress.^(16)^ Other possible explanations include the interaction between sex hormones and opioid and the hormonal fluctuations associated with the menstrual cycle.^(14,17)^ Although post-endoscopic nausea is a minor adverse effect, it may have an impact on the willingness to repeat endoscopy. Endoscopists should recognize that bidirectional endoscopy may increase post-endoscopic nausea in female patients using pethidine.

The limitation of this study is its retrospective design. A follow-up study should be performed prospectively to confirm and clarify the characteristics of adverse events after bidirectional endoscopy.

In conclusion, bidirectional endoscopy could increase hypoxia in elderly patients using pethidine and post-endoscopic nausea in female patients.

## Figures and Tables

**Table 1:** Characteristics and adverse events of colonoscopy

	Colonoscopy only	Bidirectional endoscopy	*p* value
Patients			
Number	652	550	
Age (years)	53.5 ± 13.7	51.6 ± 11.5	0.0078
Female sex (%)	361 (55.4%)	269 (48.9%)	0.0255
Indication			0.146
Diagnostic	172 (26.4%)	124 (22.5%)	
Screening	294 (45.1%)	244 (44.4%)	
Polyp surveillance	186 (28.5%)	182 (33.1%)	
Sedation			
Use of pethidine	364 (55.8%)	482 (87.6%)	<0.001
Dose of pethidine	13.3 ± 13.8	24.2 ± 13.1	<0.001
Use of midazolam	592 (90.8%)	540 (98.2%)	<0.001
Dose of midazolam	3.14 ± 1.37	3.96 ± 1.18	<0.001
Procedure			
With polypectomy	381 (58.4%)	274 (49.8%)	0.0028
Adverse events			
Hypoxia	14 (2.1%)	44 (8%)	<0.001
Nausea	2 (0.31%)	9 (1.6%)	0.0158
Abdominal pain	1 (0.15%)	1 (0.18%)	0.904
Bleeding	4 (0.61%)	0	0.066
Perforation	0	0	

**Table 2:** Univariate analysis and multivariate analysis on hypoxia

Variables	Univariate analysis				Multivariate analysis		
	Odds ratio	95% CI	*p* value		Odds ratio	95% CI	*p* value
Age	1.047	1.026–1.069	<0.001		1.061	1.035–1.087	<0.001
Sex (Female)	1.029	0.607–1.745	0.978				
Bidirectional endoscopy	3.963	2.147–7.312	<0.001		3.658	1.91–7.008	<0.001
Use of pethidine	4.692	1.859–11.84	<0.001		4.311	1.627–11.42	0.003
Use of midazolam	7.677*	0.469–125.5	0.16				
With polypectomy	2.089	1.173–3.721	0.016		1.79	0.976–3.284	0.06

*Estimated odds ratio (Haldane correction)

**Table 3:** Univariate analysis and multivariate analysis on nausea

Variables	Univariate analysis				Multivariate analysis		
	Odds ratio	95% CI	*p* value		Odds ratio	95% CI	*p* value
Age	0.993	0.946–1.04	0.754				
Sex (Female)	9.21	1.175–72.17	0.024		10.25	1.304–80.55	0.027
Bidirectional endoscopy	5.406	1.163–25.1	0.035		6.051	1.297–28.22	0.022
Use of pethidine	9.813*	0.577–166.9	0.104				
Use of midazolam	0.615	0.078–4.873	0.856				
With polypectomy	0.476	0.138–1.633	0.366				

*Estimated odds ratio (Haldane correction)
